# Surface Modification of an Absorbable Bimodal Fe-Mn-Ag Alloy by Nitrogen Plasma Immersion Ion Implantation

**DOI:** 10.3390/ma16031048

**Published:** 2023-01-25

**Authors:** Pedram Sotoudeh Bagha, Carlo Paternoster, Mehrdad Khakbiz, Saeed Sheibani, Navid Gholami, Diego Mantovani

**Affiliations:** 1 BiionixTM (Bionic Materials, Implants & Interfaces) Cluster, Department of Medicine, University of Central Florida College of Medicine, Orlando, FL 32827, USA; 2 Lab Biomaterials and Bioengineering, CRC-I, Department of Mining, Metallurgical and Materials Engineering & CHU de Quebec Research Center, Regenerative Medicine, Laval University, Quebec City, QC G1V 0A6, Canada; 3 Department of Chemical and Biochemical Engineering, Rutgers, The State University of New Jersey, Piscataway, NJ 088854, USA; 4 Division of Biomedical Engineering, Faculty of New Sciences and Technologies, University of Tehran, Tehran 14395-1561, Iran; 5 School of Metallurgy and Materials Engineering, College of Engineering, University of Tehran, Tehran 11155-4563, Iran; 6 Department of Tissue Engineering and Applied Cell Sciences, School of Advanced Technologies in Medicine, Tehran University of Medical Sciences, Tehran 14177-55469, Iran

**Keywords:** Fe-Mn-based absorbable alloy, biocompatibility, corrosion, surface modification, nitrogen plasma immersion ion implantation

## Abstract

Recently, Fe-Mn-based alloys have been increasingly catching the attention of the scientific community, because of their tunable and outstanding mechanical properties, and suitable degradation behavior for biomedical applications. In spite of these assets, their corrosion rate (CR) is, in general, too low to satisfy the requirements that need to be met for cardiovascular device applications, such as stents. In fact, the CR is not always the same for all of the degradation stages of the material, and in addition, a finely tuned release rate, especially during the first steps of the corrosion pattern, is often demanded. In this work, a resorbable bimodal multi-phase alloy Fe-3Mn-1Ag was designed by mechanical alloying and spark plasma sintering (SPS) to accelerate the corrosion rate. The presence of several phases, for example α-Fe, α-Mn, γ-FeMn and Ag, provided the material with excellent mechanical properties (tensile strength UTS = 722 MPa, tensile strain A = 38%) and a higher corrosion rate (CR = 3.2 ± 0.2 mm/year). However, higher corrosion rates, associated with an increased release of degradation elements, could also raise toxicity concerns, especially at the beginning of the corrosion pattern. In this study, The focus of the present work was the control of the CR by surface modification, with nitrogen plasma immersion ion implantation (N-PIII) treatment that was applied to mechanically polished (MP) samples. This plasma treatment (PT) improved the corrosion resistance of the material, assessed by static degradation immersion tests (SDITs), especially during the first degradation stages. Twenty-eight days later, the degradation rate reached the same value of the MP condition. Nitrogen compounds on the surface of the substrate played an important role in the corrosion mechanism and corrosion product formation. The degradation analysis was carried out also by potentiodynamic tests in modified Hanks’ balanced salt solution (MHBSS), and Dulbecco’s phosphate buffered saline solution (DPBSS). The corrosion rate was higher in MHBSS for both conditions. However, there was no significant difference between the corrosion rate of the PT in DPBSS (CR = 1.9 ± 0.6 mm/year) and in MHBSS (CR = 2 ± 1.4 mm/year). The cell viability was assessed with human vein endothelial cells (HUVECs) via an indirect metabolic activity test (MTT assay). Due to the lower ion release of the PT condition, the cell viability increased significantly. Thus, nitrogen implantation can control the in vitro corrosion rate starting from the very first stage of the implantation, improving cell viability.

## 1. Introduction

A revolution in biomedical implants started with the introduction of absorbable metallic alloys. This group of alloys needs to satisfy specific requisites, as they are supposed to support the damaged tissue, and to show a pro-active interaction with the surrounding biological environment. For this reason, appropriate mechanical properties, and simultaneously, a CR guaranteeing any local or systemic toxicity are required. In any case, they are supposed to maintain their mechanical integrity and degrade until the healing procedure is complete. Different absorbable alloys, such as Fe- [1,2,3], Mg- [4,5,6], and Zn-based [7,8,9] ones have been reported. Their properties depend on their chemical composition, microstructure, thermomechanical history, and surface state [10,11,12,13].

Pure Fe is known for its excellent mechanical properties, which are similar to those of 316 L stainless steels, namely yield strength (~150 MPa), tensile strength (~200 MPa), and maximum elongation at rupture (~40%); furthermore, pure Fe shows excellent biocompatibility features [14]. The degradation properties in in vitro and in vivo environments were assessed, showing, in general, an average CR in the range of 0.1 to 0.56 mm/year [15,16]. A targeted CR should be in the range of 0.5 to 0.8 mm/year [16]; in addition, the corrosion process should allow a gradual and uniform degradation, to maintain the mechanical resistance during the healing process; finally, the scaffold should be completely resorbed and disappear after the tissue remodeling. To increase the electrochemical performances of pure Fe (CR = 0.1 mm/year [17]), different strategies have been proposed, including alloying with elements, such as Mn [12,13,14,15,16], to decrease the alloy overall corrosion potential. The incorporation of other elements (Pd, Au, Ag) and compounds/nanostructures (Mg_2_Si, Ca_7_MgSi_4_O_16_, CNT, etc.) was also explored, to introduce corrosion-enhancing micro-galvanic effects [17,18,19,20,21,22]. A different approach consisted in accelerating the corrosion rate of Fe-C alloy by adding MnO_2_ as a catalyst promoting electron transfer [18], while forming a passive oxide layer after an immersion test, susceptible to decrease the degradation rate.

Even if Fe-Mn-based alloys show a range of suitable microstructural, mechanical, and biological properties, their average low degradation rate is still a reason of concern. For example, Fe-Mn-C-Pd [3] shows a CR = 0.21 mm/year. Other studies showed that Fe-Mn-Pd pins did not significantly degrade, even after 52 weeks of implantation [11]. The formation of passivating corrosion layers, composed by phosphates, carbonates, oxides and/or hydroxides [19], inhibits further degradation. To obtain a material composed by structural elements with different corrosion patterns, multi-phase bimodal-structured alloys (with a combination of nano- and micro-structured grains) were proposed; it was shown by Sotoudeh Bagha and colleagues [20] that such a microstructure did not reach a steady-state in an open circuit potential test and the electrochemical corrosion rate reached a value of a CR = 0.88 mm/year. However, the non-uniform corrosion behavior of Fe-based bimodal alloys needs to be controlled. Other authors showed that SPS Fe-based alloys for biomedical applications, that is Fe-Pt and Fe-Pd, had an increased corrosion rate, compared to that of pure Fe [21]: potentiodynamic tests evidenced that the introduction of other elements in these kinds of alloys, created new phases and galvanic couples, were responsible for a corrosion increase in the range of ~1.8 times (Fe-Pd) to ~7.7 times (Fe-Pt) the value corresponding to pure Fe. For Fe-Mn-Cu obtained with a traditional powder metallurgy route [22], potentiodynamic tests showed that the corrosion rate increased six times for the alloy containing 10 wt% Cu, compared to the base alloy, which showed a CR = ~0.045 mm/y. Surface modification is a well-known treatment to control the corrosion behavior and to improve the biocompatibility of the biomedical alloys [23,24]. For instance, nitrogen and oxygen PIII (N-PIII and O-PIII, respectively) treatments improved the corrosion resistance and the biocompatibility of Ti- [25] and Co-based alloys [26], respectively. Furthermore, the formation of iron nitrides (Fe_2_N and Fe_3_N, with a thickness in the range of 40–140 nm [27]) after N-PIII, significantly improves the electrochemical corrosion resistance of pure Fe in 0.9% NaCl solution [28]. In addition to the electrochemical behavior in absorbable implants, it is necessary to understand the long-term corrosion behavior with a SDIT. For instance, the formation of different species of degradation products on the surface can influence the degradation rate of Fe-Mn-based alloys [29], so it is necessary to understand the effect of the modified nitride layer on the alloy corrosion pattern. Furthermore, O-PIII on Fe improved the adhesion and proliferation of endothelial cells [30]. Furthermore, N-PIII on Ti enhanced the preosteoblast and fibroblast responses to the surface and improved the biocompatibility [31]. However, at the moment of the redaction of this paper, no studies reported the interaction of cells with N-PIII-modified SPS-fabricated bimodal multiphase Fe-Mn-based alloys.

Hence, the objective of applying the surface treatment was to control the degradation rate of the bimodal alloy during the early stages of implantation. In particular, in the present study, a bimodal Fe-Mn-Ag alloy was prepared, with the aim of studying the behavior of the alloy through a SDIT. Different pseudo-physiological solutions were considered: (1) MHBSS and (2) DPBSS. The material was investigated after mechanical polishing (MP) and after N-PIII. Complementary characterization techniques were implemented to evaluate the surface features, before and after SDIT and electrochemical tests. Finally, to evaluate the cytotoxicity of the studied conditions, cell viability tests were performed by an MTT assay using human umbilical vein endothelial cells (HUVECs).

## 2. Materials and Methods

### 2.1. Alloy Fabrication and Surface Preparation

Fe (particle size average diameter ~10 µm, 99.5% pure), and Mn powders (particle size average diameter <100 µm, ≥90%, 99.0% pure) were purchased from Merck Millipore (Burlington, MA, USA), while pure Ag (particle size average diameter ~2–3.5 μm, 99.9% pure) was purchased from Sigma-Aldrich (St. Louis, MO, USA). A planetary ball mill (NARYA MPM-2 * 250 H mode, Amin Asia Fanavar Pars, Tehran, Iran) was used to mill the powders with a nominal chemical composition of Fe-30Mn-1Ag in weight percent (ball-to-powder weight ratio of 30:1, duration of 10 h, rotation speed of 250 rpm). Then, an equal portion of unmilled powders, whose composition was approx. 70 wt.% Fe and 30 wt.% Mn, was mixed with the milled powder. The bimodal powder mixture (50% milled powder + 50% unmilled powder) was consolidated by SPS equipment (EF-20T-10, Easyfashion, Changsha, China) at 1000 °C in a graphite mold. The heating rate, holding time and pressure of the SPS were 50 °C/min and 5 min and 40 MPa, respectively. The material density of the samples was measured by a helium pycnometer, based on a gas displacement method (AccuPyc II 1340 Pycnometer, Micromeritics Instrument Inc., Norcross, GA, USA). For this purpose, samples with a thickness of 1.8 mm were cut into 4 disks with the final thickness of 0.45 mm, and they were analyzed. The pycnometer measurement volume was filled with helium gas and the sample volume was measured. Then, the density was calculated from the measured volume and the sample mass [32].

### 2.2. Mechanical Testing 

Hardness and micro-hardness tests were carried out on the polished samples, using a Rockwell B tester (RT120, Leco, St. Joseph, MI, USA) with a load of 100 kgf, and a 1⁄16 inch-diameter steel sphere. A Vickers micro-hardness tester (MMT-X7A, Matsuzawa, Akita, Japan) with a load of 50 g for 13 s was used to assess the phases’ microhardness. Each test was repeated five times on three different samples. A shear punch test was used to measure the shear strength of the samples by using a 20 kN universal testing machine (STM20, Santam, Tehran, Iran). A detailed description of the shear punch test can be found elsewhere [28]. The test was repeated on three different samples.

Specimens in the shape of disks (diameter Ø = 15 mm, thickness = 1.8 mm) were ground with a series of abrasive papers, from grit No. 240 to 7000, and rinsed in 96 vol.% ethanol (commercially pure) to avoid surface oxidation. Then, they were polished with a suspension of 1 µm-diameter diamond paste on a cloth Leco, St. Joseph, MI, USA). This surface preparation method was used to produce the MP samples. Following the polishing, the specimens were rinsed with 96% ethanol, dried with warm air (Struers Drybox-2, Copenhagen, Denmark) and then stored in a desiccator until later use.

### 2.3. Plasma Surface Modification

N-PIII was performed with a radio frequency (*f* = 13.56 MHz) inductively coupled plasma reactor system (PBII-300 Plasmionique, Varennes, QC, Canada) with a nitrogen pressure P = 10 mTorr (gas purity 99.9%). The residual pressure was P_0_ = ~3.4 × 10^−4^ mTorr; the nominal applied pulsed bias was *U_bias, t_* = −10.0 kV, corresponding to a real applied power *U_bias, r_* = −9.90 kV. Pure nitrogen (99.995%, Linde Gas, Mississauga, ON, Canada) with a flow rate of Φ = 10 sccm and an applied power *PW* = 300 W was used. The substrate bias was applied using short pulses with a duration *t_pulse_* = 10 μs with a repetition rate *r* = 1000 Hz, for a total duration *t* = 60 min. Both circular faces were ground implanted. The condition was addressed as plasma treated PT, as previously specified.

### 2.4. Surface Characterizations

A surface chemical analysis was carried out by XPS (PHI 5600-ci spectrometer, Physical Electronics, Chanhassen, MN, USA). The incident angle and residual pressure were respectively *φ* = 45° and *p* = 3 × 10^−9^ Torr. A survey spectrum covering the range of 0–1400 eV was recorded using an Al K_α_ X-ray source (1488.6 eV) at 200 W. High-resolution spectra of O1s, C1s, N1s, Fe2p and Mn2p regions were recorded with a Mg K_α_ X-ray source (1253.6 eV) at 150 W. Three samples were investigated by survey and high resolution for each condition. The curve fitting was calculated through the least square method (Gauss 80–90%-Lorentz 20–10% functions after the Shirley background correction) with PHI MultiPak^MT^ software version 9.3.0.3 (2011-12-06; copyright © Ulvac-phi, Inc., 1994–2011).

A scanning electron microscope (SEM, Quanta 250, FEI Company, Eindhoven, The Netherlands) was used for the microstructural analysis of the MP samples, after 1% nital etching. The system was equipped with a tungsten filament, with an acceleration voltage in the range 15.0–20.0 kV. The Fe, Mn and Ag distributions of the sample was assessed by electron probe microanalysis (EPMA). An electron probe (SX-10, Cameca, Gennevilliers Cedex, France) was used as the acquisition system. The probe current, and the acceleration voltage were *i* = 20 nA, *V* = 15 kV, respectively.

The static drop contact angle tests were performed to compare the relative hydrophobicity of the samples. Three different samples were measured by a drop shape analysis system (VCA Optima XE, AST Products, Billerica, MA, USA) with 5.0 μL of deionized water.

### 2.5. Electrochemical Measurements

The electrochemical behavior of the two conditions was studied by open circuit potential (OCP), electrochemical impedance spectroscopy (EIS) and potentiodynamic polarization (PDP) tests. A potentiostat (VersaSTAT 3, Princeton Applied Research, Princeton, NJ, USA) was used to carry out the electrochemical tests at a temperature T = 37 ± 1 °C in the two different pseudo-physiological solutions as above mentioned, that is MHBSS and DPBSS. Details of the solution preparation and pH adjustment were described elsewhere [33]. The ion concentration of the solutions and blood plasma are compared in Table 1. The exposed sample surface to the solution was 0.096 cm². OCP tests were carried out for a duration of 4 h, as the potential did not reach the stability in 1 h. The EIS tests were followed by OCP tests, with a frequency range from 10,000 to 0.01 Hz and by applying a sinusoidal potential perturbation of 10 mV rms. EC-Lab® v11.43 (Biologic, France) was used to analyze the equivalent circuits. PDP tests were performed according to ASTM G59 [34]. The scan rate was set at 0.166 mV/s; the scanning potential window was in the range of −0.25 V to + 0.6 V, and it was defined on the bases of the OCP. Each test was repeated on three samples per condition and analyzed with the EC-Lab software (Biologic, France). The corrosion rate (CR) was calculated based on the ASTM G59 standard [34] using Equation (1):

(1) $$\mathrm{CR}=3.27\times 10^{-3}\times \frac{i_{\mathrm{corr}}\times \mathrm{EW}}{D}$$

where CR is the corrosion rate, in [mm/year], i_corr_ is the current density [μA/cm^2^], EW is the equivalent weight, based on the oxidation of Fe to Fe^2+^ and considered to be 27.92 [g/eq] and D is the material density and considered to be 7.76 [g/cm^3^] [20]. The constant *K*_1_ = 3.27 × 10^−3^ has the measurement units of [mm·g·μA^−1^·cm^−1^·year^−1^].

### 2.6. Static Degradation Immersion Tests

Static degradation immersion tests were carried out on the basis of ASTM G31-03 [35]. Samples with a diameter Ø = 15 mm and a thickness t = 1.4 mm were weighed before the test. The degradation tests were carried out in an incubator at 37 ± 1 °C. Three samples for each condition were prepared, for a total of 12 for all of the studied conditions. Each specimen was immersed in 110 mL of solution, either MHBSS or DPBSS, filling a beaker of appropriate volume. Samples were suspended with a nylon line, so that they were approximately in the middle of the medium liquid volume. The beakers were covered to avoid occasional external contamination. The tests were carried out for a duration of 14 and 28 days, respectively. Following the end of the test, the samples were removed and ultrasonically cleaned for 5 min in a 70% ethanol solution; after cleaning, each sample was weighed with a 5-digit precision balance. Following the first ultrasound cleaning, the samples were weighed a second time; if the measured weight was the same as after the first cleaning, then no further steps were performed; otherwise, another cleaning step and another weighing session was carried out. The 70 vol.% ethanol solution used for ultrasonic rinsing was collected to analyze the degradation products both from the sample surface and from the medium precipitate. Furthermore, the waste test solution was collected and centrifuged at ν = 3000 min^−1^, to separate the supernatant from the precipitated degradation products. The precipitate-rich particles at the bottom of the bottles were mixed with a 50% pure ethanol-50% deionized water solution, vortexed and centrifuged again. Then, the solid particles were collected and desiccated in 37 °C and pressure of 30 Torr, before further analysis through SEM, EDS and XRD. The degradation rate (DR) of the specimens was calculated on the basis of Equation (2), according to ASTM G31-03 [35]:

(2) $$\mathrm{DR}=8.76\times 10^{4}\frac{M}{A\cdot t\cdot D}$$

where t is the time of immersion (h), A is the area of the sample (cm^2^), M is the mass loss (g) and D is the material density (g/cm^3^). *K*_2_ = 8.76 × 10^4^ has the measurement units of [mm·h·cm^−1^·year^−1^]; the surface characterization of the degraded specimens was conducted by SEM/EDS. To investigate the phase evolution on the surfaces of the samples after the 14-day SDIT test, a grazing incidence X-ray diffraction (GIXRD) analysis, using a PHILIPS PW1730) system, was carried out. The incidence angle, step size and time per step were 1°, 0.05 and 1 s, respectively. The machine was equipped with a Cu tube (*λ_K__α__Cu_* = 1.5418 Å).

### 2.7. Cytotoxicity Tests 

The indirect cytotoxicity assays were carried out using human umbilical vein endothelial cells (HUVECs). The alloy extract for the indirect test was prepared by incubating the alloy sample in PBS with a surface area/extraction medium ratio of 1.25 cm^2^/mL [36] in an incubator at 37 ± 1 °C and humidified atmosphere for 24 h. The HUVEC line was cultured in Dulbecco’s Modified Eagle’s Medium (DMEM) with 10% fetal bovine serum, 100 U/mL penicillin, and 100 mg/mL streptomycin, in an incubator at 37 ± 1 °C in a humidified atmosphere with 5% CO_2_. An amount of 100 µL of culture medium, containing approximately 5 × 10^3^ cells, was seeded into a 96-well plate and incubated for 24 h to allow for thecell attachment. Then, for the indirect tests, the medium was replaced with the alloy extract and incubated for 24, 72 and 120 h, respectively. The concentrations of Fe, Mn and Ag ions after 24 h incubation was measured by inductively coupled plasma (ICP-OES, 5110 SVDV, Agilent Technologies, Santa Clara, CA, USA). Following the incubation, the cell metabolic activity was assessed using an MTT ((3-(4,5-Dimethylthiazol-2-yl)-2,5-diphenyltetrazolium bromide) assay: 10 µL of MTT solution (0.5 mg/mL) was added to each well after incubation at 37 °C for 4 h. Dimethyl sulfoxide (DMSO) was added to dissolve the formazan crystals and then the absorbance of the solution was measured using a microplate reader at the wavelength λ = 570 nm.

### 2.8. Statistical Analysis

Where not specified, all data are presented as mean with standard deviation (mean ± SD) for at least three samples. A statistical analysis was performed by one-way ANOVA and significance was considered for *p*-values lower than 0.05 via a Tukey test.

## 3. Results and Discussion

### 3.1. Microstructure and Mechanical Properties

**Figure 1:**
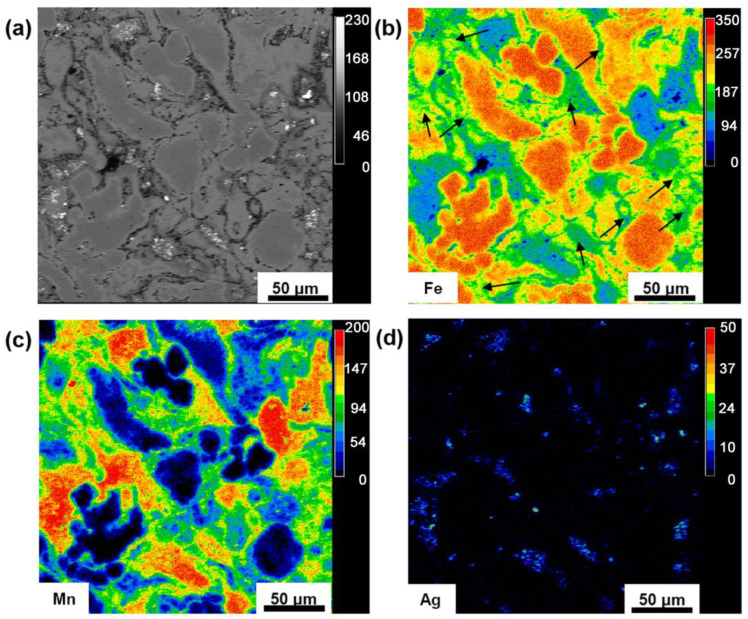
The microstructure of the consolidated sample (**a**) backscattered electron microscopy (BSE) and microprobe analysis (EPMA); (**b**) Fe (arrows represent the γ-FeMn phase); (**c**) Mn; and (**d**) Ag.

A typical element distribution of the alloy analyzed by EPMA is reported in Figure 1. Figure 1a shows a secondary electron image of a section of the studied material; element distribution of Fe, Mn and Ag are illustrated in Figure 1b–d. The milled powder formed the fine structure among the coarser Fe and Mn particles (arrows in Figure 1b). Following 10 h of milling, mechanical alloying formed γ-FeMn phase and a small amount of Ag remained in the structure without any reaction [37]. Therefore, the sample contained α-Fe, α-Mn, γ-FeMn phases and Ag-rich particles, contained in the γ-FeMn phase. The average particle size of the milled zone (γ-FeMn) and its average crystallite size were 23 ± 19 μm and 154 ± 70 nm, respectively [20]. The average measured relative density was 95 ± 1%. The mechanical properties of the consolidated sample are represented in Table 2. The micro-hardness of the different phases after etching were measured separately. The micro-hardness of α-Fe, γ-FeMn and α-Mn were H_α-Fe_ = 103 ± 13 Hv, H_γ-FeMn_ = 556 ± 77 Hv and H_α-Mn_ = 515 ± 91 Hv, respectively. This confirmed the heterogeneous structure with multiple micro-hardnesses, while the bulk hardness of the sample was H_alloy_ = 96.5 HRB, corresponding to HV_alloy_ = ~220 HV, according to ASTM E140-12B [38]. The difference among the phases could make a composite effect between α-Fe as a softer phase, and γ-FeMn and α-Mn as harder phases, which could combine good strength and ductility. Furthermore, the shear strength and shear strain of the sample were measured by a shear punch method that is a common mechanical testing method for thin section samples produced by powder metallurgy [38]. The measured mechanical properties were the shear yield strength (τ_Y_), ultimate shear strength (USS) and ultimate shear strain (A, %); they were τ_Y_ = 361.5 ± 22 MPa, USS = 420.3 ± 35 MPa and A = 74 ± 12%, respectively. The ultimate shear strength of the bimodal alloy showed a 38% improvement, compared to the micro-structured sample with the same composition [20]. This could be explained with the trend formalized with the Hall–Petch relationship, which indicated that for the finer grains, the dislocation mobility was limited, so that the grain refinement improved the mechanical strength [39]. Moreover, the shear strain was improved by 155%, compared to the nano-structured material [20]. This behavior was attributed to the higher work hardening ability of the micrometer-sized grains [40]. For the pure shear strain applied to the sample, if the von Mises criterion is applied, there is a correlation between the tensile stress.

(σ) and shear stress (τ), according to $\sigma =\sqrt{3}\tau$ [41]. This allowed for the assessment of the ultimate tensile strength as UTS = 722 MPa. Similarly, it was possible to estimate the tensile strain (ε) from the shear strain (γ), which gave $\varepsilon =\left(1/\sqrt{3}\right)\gamma$, so the tensile strain was equivalent to 38%. The mechanical property values were, in summary, comparable to those of a commercial stent material, similar to 316 L stainless steel (ultimate tensile strength UTS = 490 MPa and maximum tensile strain A = 40%) [42].

### 3.2. Surface Chemical Composition

**Figure 2:**
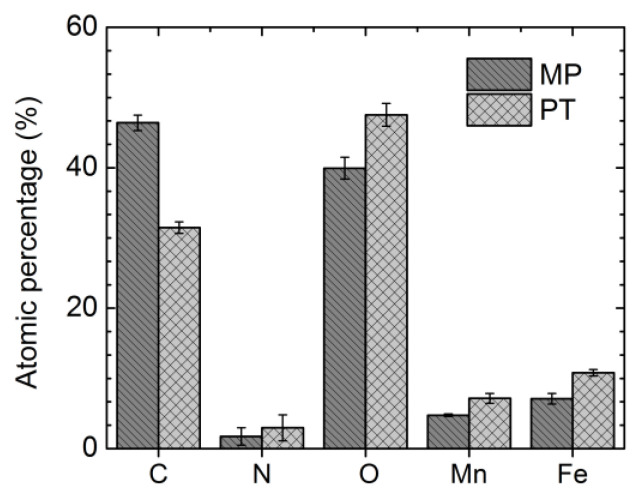
Surface element concentration for the MP and PT conditions.

**Figure 3:**
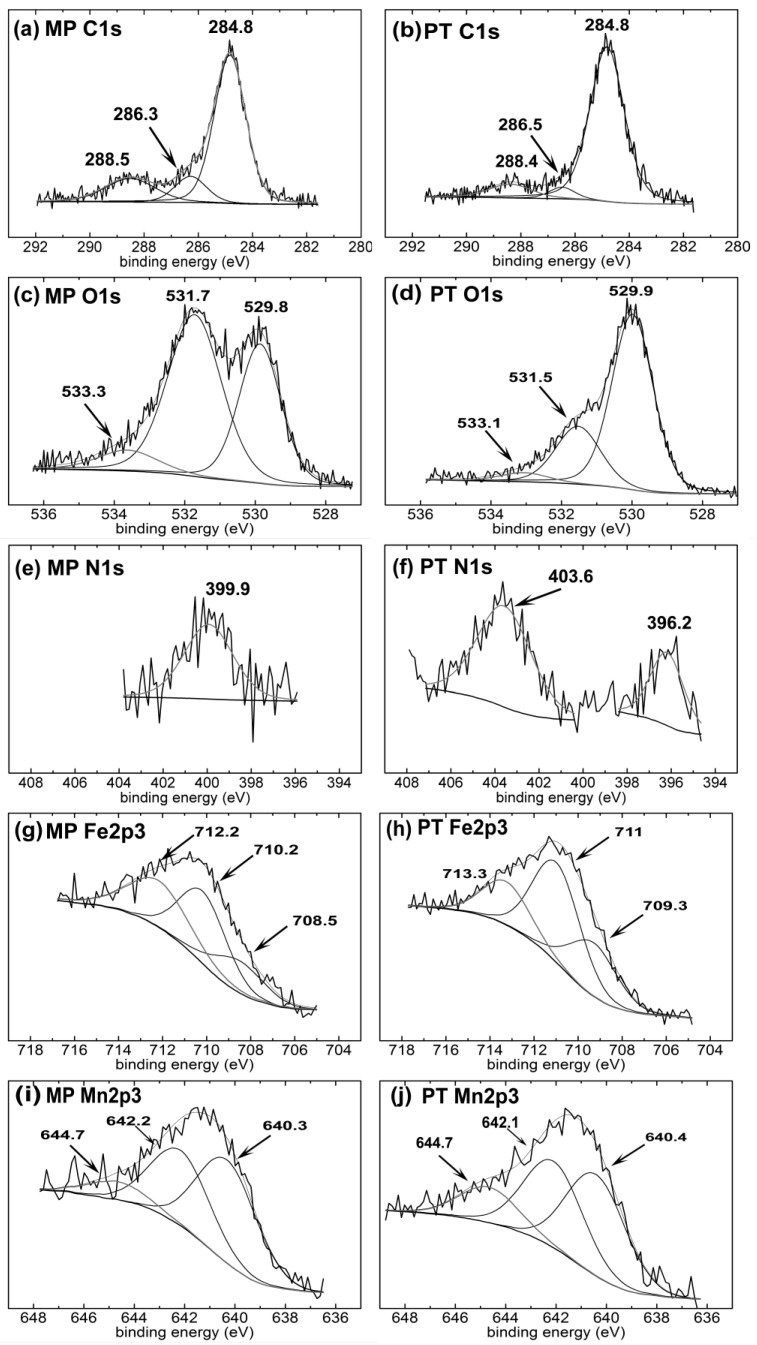
XPS high resolution deconvolutions for MP (**a**) C1s, (**c**) O1s, (**e**) N1s, (**g**) Fe2p3, (**i**) Mn2p3; and for PT (**b**) C1s, (**d**) O1s, (**f**) N1s, (**h**) Fe2p3 and (**j**) Mn2p3.

Samples were analyzed before and after the ion implantation process, by XPS survey and high resolution (Figure 2 and Figure 3, respectively) scans. The amount of C decreased from 51.1 to 34.2 at.% after the plasma treatment. Organic contamination on the surface and possible adsorbed C during polishing with ethanol, could be the main reason for a higher C detection before the PIII treatment. However, the amounts of O, N, Fe and Mn increased from 40.7, 1, 4.7 and 2.4 at.% to 50.3, 3.7, 8.6 and 3.2 at.%, respectively, after PIII. The high amount of O in the MP sample could be attributed to the formation of oxides during mechanical polishing; in addition, the oxide formation could be ascribed to the PIII treatment because of the presence of desorbed oxygen molecules in the chamber: Li et al. [43] detected, for example, a 70 nm oxide layer on the surface of steels after N-PIII, for the same reason. The small amount of N for the MP condition could arise from the adsorbed N on the surface roughness and in correspondence with the grain boundaries [44]. The general decrease of the surface contaminants, that is the carbon species, could be the reason for the detection of higher amounts of Mn and Fe after the PIII treatment.

Table 3 presents the binding energy values, full width at half maximum (FWHM), the area and fitting parameters of the peaks (percentage of the Gaussian contribution, and chi-square (χ^2^)) for C1s, O1s, N1s, Fe2p3 and Mn2p3 spectra of the samples. The C1s spectra (Figure 3a,b) showed three peaks at 284.8 ± 0.1 eV, 286.3 ± 0.1 eV and 288.4 ± 0.1 eV for both investigated conditions. The peak related to the hydrocarbon compounds is a well-known reference peak for C1s at 284.7 eV [45]; its surface area was 69.9 and 85.7% for the MP and PT samples, respectively. It has been observed that even under a vacuum condition, the carbon contamination, due to the presence of CO, CO_2_ and CHO groups, takes place [46], and in the present case, the carbon reaction with the surface was increased after the PIII treatment. One possible mechanism could be the interaction of CO and CO_2_ with the surface metallic oxide, e.g., iron oxide, and consequently the oxy-hydrocarbons formed on the surface [46]. The peak at 286.3 ± 0.1 eV could be associated with the presence of C–OH, C–O–C, C–O and C–N [47,48]; the area attributed to this energy band decreased from 12.3 to 4.8% after plasma treatment. The C1s contribution at 286.3 eV [49] could be attributed to the ethoxy species, such as ethanol; for the polishing of the samples was carried out in ethanol, the decrease of the C1s peak area at 286.3 ± 0.1 eV could be the result of the ethoxy species removal after the PIII treatment. Similarly, the peak area of C1s at 288.4 ± 0.1 eV, attributed to the carbonate species [46,47], decreased from 17.8 (MP condition) to 9.5 at.% (PT condition).

The O1s spectra of the studied conditions, as shown in Figure 3c,d, were formed by three peaks, respectively, at 530 ± 0.2 eV (O1), at 531.6 ± 0.1 eV (O2) and at 533.2 ± 0.1 eV (O3). They corresponded, in succession, to metal oxides, metal hydroxides or defective oxides and absorbed water [50,51]. For the MP condition, the areas attributed to O1 and O2 were 54.7% and 8.2%, respectively, while for the PT condition they were 25.3% and 4.4%, respectively. This decrease indicated that the O2 and O3 related species were removed partially from the surface after the PIII treatment. It should be noted that OH^−^ and CO_3_^2−^ groups had a peak overlap [52], hence the peak at approximately 531.6 eV could be ascribed to an unstable carbonate, formed by the exposure of the specimen to atmospheric CO_2_ [53]. Moreover, the peak area corresponding to O1, increased from 37.1 to 70.3% for the MP and PT samples, respectively; this trend showed the formation of a higher amount of metal oxides on the PT sample. The interaction of CO and CO_2_ with Fe formed a thin layer of FeO, which would be covered with an uneven oxy-hydrocarbon layer [46]. Hence, this could be the reason for a higher metal oxide formation after the PIII treatment.

The N1s spectra (Figure 3e,f) consisted of different peaks before and after the treatment. The MP sample showed a peak at 399.9 eV, corresponding to the atmospherically adsorbed N_2_ and oxidized nitrogen [54,55,56]. Moreover, the PT samples showed two peaks at 396.2 and 403.6 eV, corresponding to metal nitride [54,57] and N–O bonding [47,58], respectively, showing that a metallic nitride layer was formed during the N-PIII on the PT sample. Nitrogen atomic distribution along the depth of the M50 steel was reported to be in the range of 4–50 nm after N-PIII; the treatment increased the nano-indentation hardness and consequently improved the wear resistance [59]. Thus, the nitrogen implantation chemistry modification at the nanometric scale also affected the electrochemical behavior of the surface; this topic is discussed in detail in the following Section 3.3.

The peak identification of the transition metals, such as Fe and Mn, by XPS high resolution, faced several challenges according to the shake-up, plasmon loss and multiplet splitting phenomena. For instance, high spin Fe^2+^, Fe^3+^, Mn^2+^, Mn^3+^ and Mn^4+^ exhibited multiplet splitting needing careful curve-fitting on the 2p spectra. Biesinger et al. [51] applied a careful approach to fit the curves of a series of transition metals, based on the literature and practical experiments. In the present work, the Fe2p3 spectra of the MP sample contained three peaks of 708.5, 710.2 and 712.2 eV (Figure 3g), which were attributed to FeO, Fe_2_O_3_ and FeOOH, respectively [51]. Furthermore, the Fe2p3 peak at 710.2 eV could be related to iron carbonate [53], as the corresponding binding energies of O1s and C1s in iron carbonate were detected, as described earlier at 531.7 and 288.5 eV, respectively. Figure 3h shows that the Fe2p3 peaks were shifted after the PIII treatment to 709.3, 711 and 713.3 eV, and that could be attributed to Fe(III), which could bond with either nitrogen or oxygen [60] and Fe_3_O_4_ [61], while the iron nitride binding energy was reported to be in the range of 707.2–708.8 eV [54,62,63,64]. One possible mechanism responsible for the peak shift can be the formation of mixed iron nitride/iron oxide on the surface, then a thin layer of Fe_3_O_4_ was formed on the top of this mixed nitride/oxide layer [65]. Thus, the Fe2p3 peaks could be attributed to the binding of iron to either nitrogen or oxygen atoms.

The Mn2p3 spectra (Figure 3i,j) contained three peaks at 640.3 ± 0.1, 642.1 ± 0.1 and 644.7 ± 0.1 eV, which could be attributed to Mn^2+^, Mn^3+^ and Mn^4+^, respectively, indicating the formation of MnO, Mn_2_O_3,_ MnO_2_ and Mn(OH)_x_ for both conditions [51,66]. However, the binding energy of manganese nitride was considered to be, by Liu and colleagues, E = 640.7 eV [67], so the overlap of these energy bands made it complicated to quantify the amount of manganese oxide and manganese nitride formed after the plasma surface modification.

The contact angle was θ = 75.8 ± 1° for the MP condition and θ = 87.6 ± 1° for the PT condition. This implies a higher hydrophobicity for the PT condition. Many researchers observed the hydrophobicity of the biomaterial surfaces after the PIII process [24,68,69]. This phenomenon could actively affect the corrosion behavior; for example, a higher hydrophobicity of the PT sample could hinder the electrolyte penetration and help improve the corrosion resistance [70]. Furthermore, greater amounts of O and N after treatment (Figure 2), contributed to greater amounts of the surface oxide/nitride layer on the PT sample, thereby decreasing the Fe ion release in the biological solutions [71]. Moreover, the hydrophobic surfaces decreased the cell attachment and growth [72], which was beneficial to avoid the proliferation of smooth muscle cells inducing in-stent restenosis [73]. However, it should be noted that the hydrophobic surfaces increased the platelet adhesion and induced thrombosis [74], so before clinical use, the thrombogenicity of the surface should be finely tuned in case of targeted cardiovascular stent applications.

### 3.3. Electrochemical Corrosion Behavior

**Figure 4:**
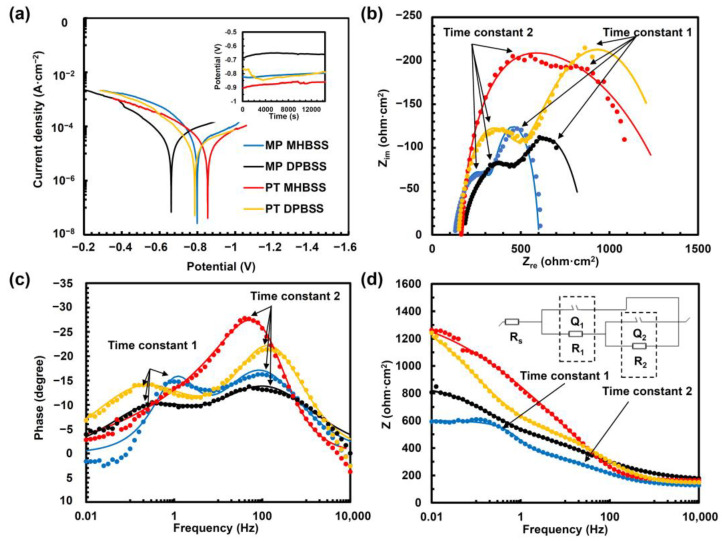
Electrochemical test results of the investigated conditions in MHBSS and DPBSS: (**a**) potentiodynamic polarization tests (inset shows the open circuit potential evaluation); (**b**) Nyquist diagram and the fitted model (lines); (**c**,**d**) Bode diagram of the samples with the fitted model (lines) (inset in (**d**) shows the equivalent circuit for the studied conditions). Time constants 1 and 2 are indicted by the arrows in low (1 Hz) and middle (100 Hz) frequencies.

Different ions, which are common in a biological environment, could affect the corrosion behavior of biomedical alloys [33,75]; for this reason, two different media, that is MHBSS and DPBSS, were used in the present work to perform electrochemical corrosion tests. The results of the electrochemical tests are presented in Figure 4 and Table 4. The PT condition showed a higher instability during the test, which could be related to more surface defects induced during the PIII treatment (Figure 4a inset). Y. Jirásková et al. [76] showed that N-PIII caused a 10% lattice expansion on the austenitic stainless steel, which consequently increased the surface defects. Furthermore, a higher active potential was observed for the PT sample in both media. As DPBSS contained a higher phosphate concentration, a phosphate passive layer could have formed on the sample surfaces of both condition surfaces. This layer protected the surface during OCP and shifted the potential to fewer active values, compared to the samples immersed in MHBSS. Hence, the corrosion potentials for the MP condition were C_P_ = −0.8 V (MHBSS) and C_P_ = −0.66 V (DPBSS), while for the PT condition, the potentials were C_P_ = −0.85 V (MHBSS) and C_P_ = −0.78 V (DPBSS). This more active potential in MHBSS was in agreement with the results of the OCP values for pure Fe, in comparison to DPBSS [33], because the stability of the passive carbonate and/or hydroxide layer in MHBSS could be influenced by the adsorption/desorption of Cl. Polarization, Nyquist and Bode diagrams are presented in Figure 4b–d. The PT condition showed a lower corrosion current density and corrosion rate, compared to the MP sample in both media. Furthermore, the corrosion current density i_C_ and the corrosion rate CR of the studied conditions were lower for the immersion in DPBSS: MP and PT i_C_ decreased from 26.3 ± 4.1 to 16 ± 11.3 µA/cm^2^ in MHBSS and from 18 ± 16.5 to 15.9 ± 2.2 µA/cm^2^ in DPBSS. Thus, a higher corrosion rate was observed for the MP condition (CR = 3.2 ± 0.2 mm/year for MHBSS and 2.4 ± 1.7 mm/year for DPBSS), compared to the PT condition (2 ± 1.4 for MHBSS and 1.9 ± 0.6 mm/year for DPBSS).

One reason for the corrosion resistance improvement could be the formation of the expanded austenite phase (γ_N_), which is a nitrogen supersaturated metastable phase with a preferred (111) plane orientation [77]. As (111) showed a high planar density, it decreased the atomic dissolution and enhanced the corrosion resistance [78]. However, this phase was unstable and could decompose into ferrite [79], which showed a higher corrosion rate, compared to austenite [80]. The decomposition of expanded austenite was dependent on austenite stabilizing elements, such as Mn [81]; therefore γ_N_ could be decomposed into ferrite in α-Fe enriched parts of the sample. Another reason could be attributed to the formation of nanometric surface grains after PIII, so that they decreased the galvanic corrosion, according to the homogeneous chemical composition of the surface [82]. Moreover, different ion concentrations of the test medium affected the polarization test. For instance, the higher amount of carbonates in MHBSS could form insoluble layers of FeCO_3_ [83] and MnCO_3_ [84], which could hinder the further dissolution of the sample and decrease the corrosion rate in MHBSS, compared to DPBSS.

The impedance behavior of the samples was electrically modeled as two porous layers with two different time constants (Figure 4b–d (inset in Figure 4d shows the equivalent circuit) and Table 4). R and Q represented the electrical resistance and the capacitance of the corrosion layer. Q was a constant phase element and its impedance (Z_Q_) could be defined, according to Equation (3) [85], as:

(3) $$Z_{Q}=Q_{0}{\left(j\omega \right)}^{-n}$$

where Q_0_ was a constant, j was the imaginary unit, ω was the angular frequency and n was a coefficient between 0.5 and 1. For n = 1, Z_Q_ represented the impedance of an ideal capacitor; for n < 1, the equivalent circuit showed the non-ideal capacitance due to the inhomogeneity of the surface. The solution resistance (R_S_) between the sample and the reference electrode was R_S_ = 176.7 ± 24 Ω. The charge transfer process at the interface of the porous outer layer and the electrolyte was described by R_1_Q_1_ (time constant 1, semicircle in Figure 4b). R_1_ was the electrical resistance of the porous outer oxide layer, while Q_1_ was its capacitance. R_1,_ for the samples, was immersed in MHBSS, and increased from 256 ± 9.9 to 673.4 ± 61 ohm·cm^2^ after the PIII treatment. It should be noted that after the plasma treatment, the defective hydroxide or oxide, as evidenced by XPS high resolution O analysis, was removed from the surface (Figure 3c,d and peak2 of O in Table 3) while the metal oxynitride was increased, hence R_1_ for the PT condition was higher than R_1_ for the MP condition in MHBSS. Q_1_ decreased from 115.7 ± 13 µF·cm^−2^·s^n^ (MHBSS), and 65.2 ± 7.6 (DPBSS) for the MP condition, to 30.1 ± 5.9 µF·cm^−2^·s^n^ (MHBSS) and 35.2 ± 2.4 (DPBSS) for the PT condition, while there was no significant change in the n values: only for the PT + MHBSS condition, it was increased to 0.9 ± 0.1; this could be associated with the presence of an outer oxide layer that shifted to a nearly ideal capacitance, thus decreasing the current flow [86] and acting as a barrier against the solution penetration.

R_2_ and Q_2_, in parallel, corresponded to the resistance and the impedance of the inner oxide/nitride layer (time constant R_2_Q_2_, semicircle in Figure 4b). The degradation layers of the Fe-based alloys had two porous oxide layers [1]. The inner oxide layer was covered by an outer ferrous hydroxide layer. The oxide layer and hydroxide layer acted as a barrier layer against the cation exchange. However, due to the low thickness of the PIII layer, mainly formed by iron nitrides, it was difficult, in the present case, to distinguish the effect, in terms of the electrochemical cation exchange, of the oxide inner layer from the PIII nitride layer, so they were modeled here as a general inner layer (R_2_Q_2_). This model was in accordance with the obtained R_2_ from the data elaboration; in particular, for the MP condition, DPBSS R_2_ = 311.1 ± 89.5 ohm·cm^2^, while MHBSS R_2_ = 235.1 ± 11 ohm·cm^2^. Moreover, the PT samples showed an increased resistance of 921.6 ± 3.5 and 1547 ± 578 ohm·cm^2^ for the DPBSS and MHBSS immersion, respectively. This trend could be related to the formation of iron nitride after PIII on the surface, as they showed a higher corrosion resistance, compared to pure Fe and Mn [87,88,89]. N-PIII on austenitic stainless steel, also proved the higher corrosion resistance and pitting resistance by stabilizing the passive film formation because of the N reduction in the presence of H^+^ [90]. Furthermore, nitrogen supersaturation enhances the diffusion of N and the formation of protective compounds, such as NH_3_ and NH^+^_4_ and inhibit localized corrosion [91].

Moreover, Q_2_ increased from 220 ± 9.2 µF·cm^−2^·s^n^ and 1454 ± 148 µF·cm^−2^·s^n^ for the MP condition in MHBSS and DPBSS, to 946 ± 79 µF·cm^−2^·s^n^ and 1637 ± 28.7 µF·cm^−2^·s^n^ for the PT condition in MHBSS and DPBSS, respectively. However, the values of n_2_ of the MP condition decreased from 1 ± 0.1 and 0.6 ± 1 to 0.6 ± 0.3 and 0.5 ± 0.1 for PT immersed in MHBSS and DPBSS, respectively. This could be attributed to a more heterogeneous surface from the chemical point of view, after the PIII treatment. Therefore, the increase of R_1_ and R_2_ contributed to the higher corrosion resistance for the PT condition. The reduced χ^2^ (χ_ʋ_^2^, observed experimental variance divided by the theoretical variance) for the impedance fitting was 0.25 for the MP + MHBSS condition, 0.12 for the MP + DPBSS condition, 0.74 for the PT+ MHBSS condition, and finally 0.1 for the PT + DPBSS, condition, respectively. Values of χ_ʋ_^2^ lower than 1 were due to the presence of a small amount of noise in the raw data but the Nyquist and Bode curves were well fitted; two peaks in the phase and the impedance change of the Bode diagrams, Figure 4c,d, correspond to the two semicircles (time constants; parallel R and Q components) in Figure 4b. The time constant (τ) was measure by Equation (4) [92], as:

(4) τ=RQ

which shows how long the capacitor takes to charge or discharge through the electrochemical processes. Time constants 1 and 2 are indicated by the arrows in low (1 Hz) and middle (100 Hz) frequencies. There are no significant changes in τ_1_ among the samples while τ_2_ increased from 0.027 ± 0.003 s and 0.02 ± 0.026 s for MP + MHBSS and MP + DPBSS, to 1.46 ± 0.32 s PT + MHBSS and 1.51 ± 0.25 s PT + DPBSS, respectively. This is in agreement with the increase of R2 and Q2, according to the formation of the oxide/nitride layer on the PT sample.

**Figure 5:**
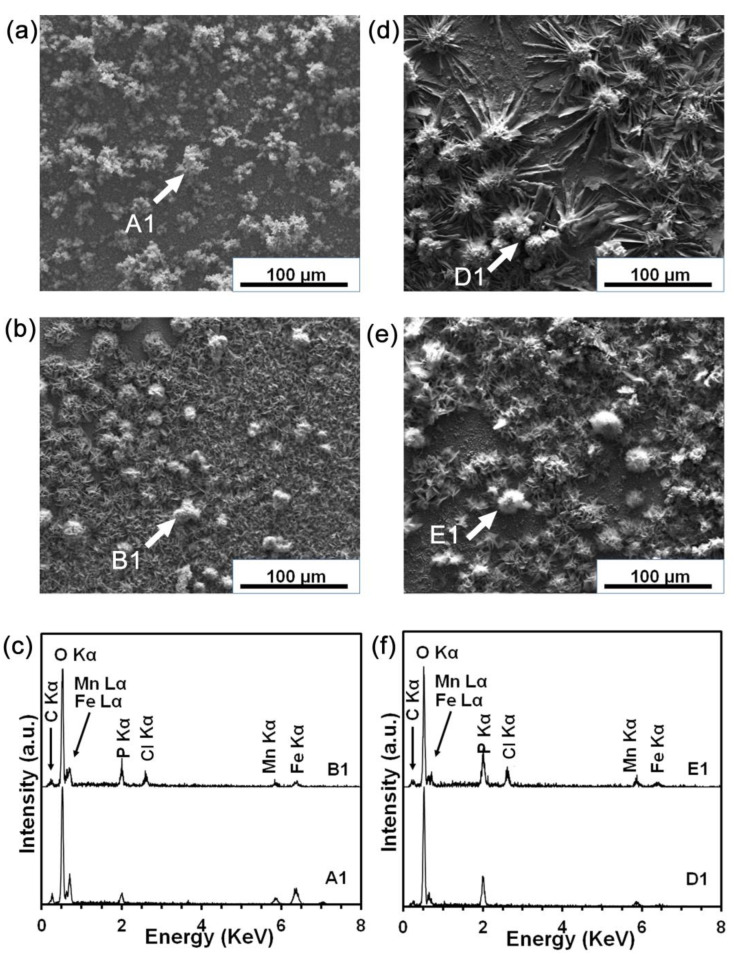
Surface morphology after the potentiodynamic polarization tests in MHBSS for (**a**) MP and (**b**) PT conditions; (**c**) EDS spectrum for A1 and B1. Surface morphology after the potentiodynamic polarization tests in DPBSS for (**d**) MP and (**e**) PT conditions; (**f**) EDS spectrum for D1 and E1.

The surface morphology after the potentiodynamic test in DPBSS and MHBSS showed significant differences (Figure 5a,b,d,e). The EDS analysis evidenced the presence of O and P on the surface of all of the studied conditions. This could be associated to the formation of passive layers. As evident from all of the reported spectra (Figure 5c,f), P and O could be associated with the formation of phosphate passive films on the surface. These precipitates could hinder the ionic exchange and consequently block further material dissolution [33]. As DPBSS contains more P than MHBSS, flower-shaped phosphate precipitations were formed on the surfaces immersed in DPBSS (Figure 5d,e).

Nevertheless, the homogeneous fine dense precipitations on the PT condition in MHBSS (Figure 5b) could be the reason for the higher R_1_, compared to the MP condition (Table 4), thereby suggesting a more uniform corrosion behavior.

The only difference between the chemical composition of the corroded MP and PT conditions was a small amount of Cl^−^ that was detected on the surface of the MP sample surface. The pitting could be inhibited when the amount of P ions was higher than the Cl^−^ ions [93]; therefore, the sample was more prone to pitting in MHBSS. Nitrogen could pass through on the surface as NH_x_, and could be dissolved as NH_4_^+^ [94]. The presence of negatively charged nitrogen (N^δ−^) at the oxide/solution interface could inhibit the pit initiation by suppressing the Cl^−^ adsorption; consequently, the corrosion rate was decreased, and a uniform corrosion mechanism was triggered. Moreover, the cathodic nitrogen dissolution could increase the pitting resistance by decreasing the pH drop in the pits, by forming NH_4_^+^, as in Equation (5) [95]:

(5) $$N+4H^{+}+3e^{-}⇄NH_{4}^{+}$$

However, the following reactions (dissolution–precipitation) could take place and cause further corrosion (Equations (6) and (7)) [96]:

(6) $$Fe^{2+}+2Cl^{-}⇄FeCl_{2}$$

(7) $$FeCl_{2}+Cl^{-}⇄FeCl_{3}+e^{-}$$

The deposition of a mixture of Fe oxy-hydroxides, in which some Cl was also present, increased the pitting resistance of the surface [97]. In fact, improvement of the pitting resistance was proved by N-PIII to 316 stainless steel by 36%, according to the formation of iron nitrides [98].

### 3.4. Static Degradation Tests Results 

**Figure 6:**
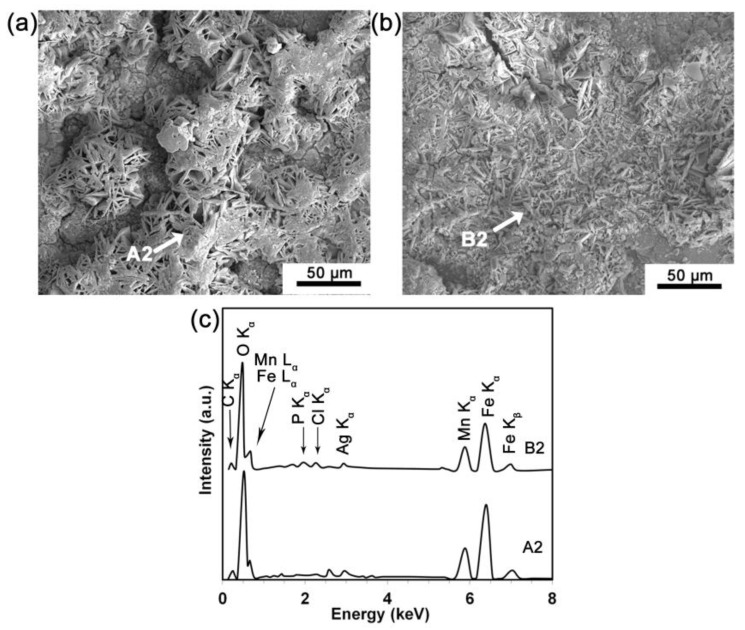
The surface of the degraded samples after a 14-day immersion in MHBSS (**a**) MP and (**b**) PT sample, and (**c**) EDS for points A2 in (**a**) and B2 in *(***b**).

The surface morphology and chemical composition of the MP and PT conditions after 14-day SDITs in MHBSS are presented in Figure 6. The surfaces of both conditions, after the test, were covered by a layer of degradation products, containing Fe, Mn, O, Cl, P and C. The chemical compositions were compatible with that from a layer containing phosphates and carbonates of Fe and Mn, as already found [29,97]. This implies the formation of a degradation product layer on the PT condition, corresponding to the fine precipitations in Figure 5b, that were not removed with the cleaning. A punctual EDS analysis of the surfaces in Figure 6a,b (points A2 and B2, respectively) were compared in Figure 6c; they showed similar composition profiles, but the different thickness and morphology of the precipitate layers for the two conditions could be the main reason of the different corrosion behavior of the samples. Following the O and N implantation, the formation of new phases, in the form of nanocrystalline oxides and nitrides [99,100], took place. The fine-structure and uniform thickness surface layer played a role in the nucleation and growth of a thinner and homogeneous degradation product film, compared to the coarser one obtained for the MP condition.

**Figure 7:**
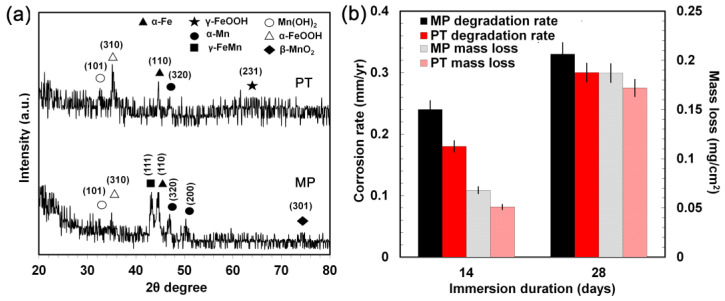
(**a**) Phase attribution for the MP and PT condition surfaces after the 14 day SDITs (GIXRD patterns); (**b**) static degradation rate and mass loss for the MP and PT conditions after 14 and 28 days of immersion.

To analyze the phase composition of the degradation products after the SDITs, the GIXRD was performed (Figure 7a). On the corroded surface of both conditions α-Fe, α-FeOOH, α-Mn and Mn(OH)_2_ were detected. However, β-MnO_2_ was found on the surface of the MP sample, while γ-FeOOH was mainly found on the PT sample. As discussed earlier, N^δ−^ at the interface of the oxide/solution may suppress the negative ion adsorption, such as Cl^−^ and OH^−^, and affect the formation of different iron hydroxides [101]. When absorbable metals are used in biomedical applications, the absorption of the corrosion products is studied, to assess the risk of local degradation product accumulation. Feng et al. [102] investigated plasma nitride iron stents through in vivo tests and detected yellowish accumulated corrosion products on the implanted stent surface. The corrosion layer contained iron nitride that was not fully absorbed, even after 12 months of implantation.

On the contrary, in the present work no relevant presence of any nitride phase was detected: this was compatible with the supposed limited thickness of the surface modified layer obtained with PIII. Consequently, there would be a lower possibility of corrosion product accumulation, because the limited amount of nitride-formed debris would be slowly absorbed or disposed of by the macrophages. The degradation rate of the MP and PT conditions increased from 0.24 and 0.18 mm/year (at day 14) to 0.33 and 0.3 mm/year (at day 28), respectively (Figure 7b). Thus, the degradation rate of the PT condition was lower at the beginning and then reached approximately that of the MP condition after 28 days. Similarly, the mass loss of the MP and PT conditions showed a marked difference after 14 days (0.068 and 0.051 mg/cm^2^, respectively) while after 28 days, the mass loss increased to 0.187 and 0.172 mg/cm^2^ for the MP and PT samples, respectively. The results of the electrochemical (Table 4) and static degradation tests (Figure 7b) showed the same trend, that is a lower corrosion rate for the PT condition. The lower degradation rate of the static test was attributed to the formation of the corrosion products, such as iron hydroxide [33], on the surface of the samples, whose effect was to inhibit the medium penetration toward the substrate.

**Figure 8:**
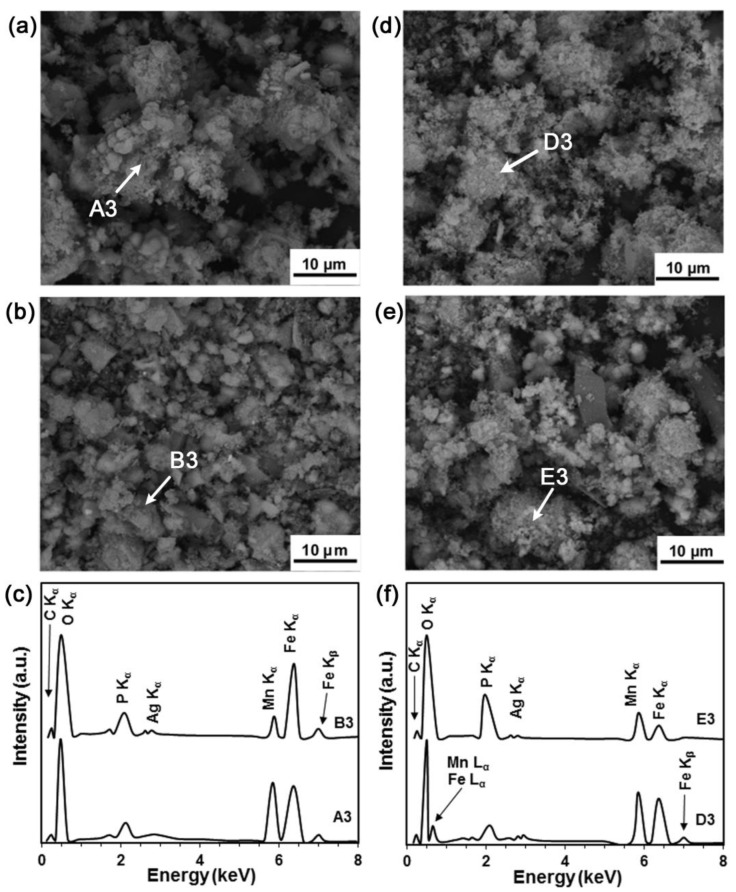
Degradation products after 14 days of immersion in MHBSS from (**a**) the MP and (**b**) PT conditions; (**c**) EDS for points A3 in (**a**) and B3 in (**b**). Analysis of the powders collected from the bottom of the solution, for (**d**) the MP and (**e**) PT conditions; (**f**) EDS for points D3 in (**d**) and E3 in (**e**).

Figure 8 shows the degradation products obtained from the sample surfaces (Figure 8a,b) and collected from the bottom of the test solution (Figure 8d,e), after the 14-day SDITs. There was no significant difference between the chemical composition of the degradation products after the MP and PT ultrasound cleaning (Figure 8c). However, the PT condition degradation products showed, qualitatively, a more uniform size, while the MP condition degradation products were more prone to agglomeration (larger than 10 μm). In comparison, the morphology of the particles collected from the bottom of the solution after degradation did not show any differences (Figure 8d,e).

### 3.5. Cell Viability

**Figure 9:**
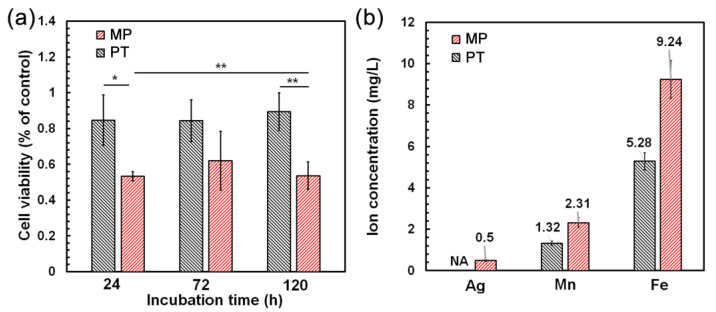
(**a**) Cell viability of the samples after 24, 72 and 120 h of incubation with HUVECs (n = 3, *: *p* < 0.05; **: *p* < 0.01) and (**b**) ion release after 24 h immersion of the samples (NA: No Ag detected).

Figure 9a shows the cell viability of the samples through an indirect MTT assay. The metabolic activity of the cells improved to 84% for the PT sample from 53% for the MP sample after 24 h of incubation. These values were similar for 72 and 120 h of incubation, this outcome showed an acceptable cytocompatibility (more than 75% of the control [36]) for the PT condition.

One major factor affecting the cytotoxicity, was the amount of released metal ions [103], so the higher corrosion resistance of the PT sample could have reduced the toxicity. It has been proved that a higher corrosion resistance of nitrogen-containing steels, and consequently a lower ion release, enhanced not only the cell viability, but also the cell adhesion and hemocompatibility of the alloys [104,105,106,107,108]. Moreover, the negative charge of the surface, the higher hydrophilicity and surface roughness of the N-PIII treated Ti alloy, significantly increased the fibronectin adhesion to the surface, which promoted the Ca^2+^ formation and human bone marrow mesenchymal stem cell adhesion [109]. Consequently, higher cell adhesion enhanced the bone implant contact. Thus, the nitrogen implantation can improve the biocompatibility of the metallic surface. Another factor that was observed in the N-PIII treatment of silk and zirconia, was a better spreading of the cells according to the surface modified functional groups [110,111]. However, it did not have a negative impact on the immune system response (macrophages). However, there is lack of study on the N-PIII of biodegradable iron implants and more in vitro and in vivo investigations are required.

To investigate the ion release over 24 h in the samples, ICP-OES was performed (Figure 9b) on the corresponding extracts. Ag was not detected for the PT condition, while a concentration c_Ag_ = 0.5 mg/L was released from the MP sample. This could be one reason for the higher cell viability of the PT sample. It has been shown that less than 0.05 mg/L of Ag ion release could slightly decrease the cell viability of the HUVEC line [112,113]. Furthermore, the amounts of ions released from the MP and PT samples decreased from 9.24 to 5.28 mg/L for Fe and from 2.31 to 1.32 mg/L for Mn, respectively. A high toxicity was observed for the fibroblast, smooth muscle and HUVEC lines due to the high amount of Mn ions [114,115]; pure Mn showed a significantly higher toxicity, compared to the Fe-Mn alloy [1]. Hence, as the MP condition contained a pure Mn phase, the cell viability decreased significantly. The ion release resulted in agreement with the electrochemical and static corrosion tests, which showed a higher corrosion rate for the MP condition. As a result, the higher cell metabolic activity was achieved after the PIII treatment.

## 4. Conclusions

In this study, the influence of N-PIII on the corrosion behavior and biocompatibility of an absorbable bimodal Fe-Mn-Ag alloy was investigated. The microstructure of the alloy was found to present different phases (α-Fe, α-Mn and a mechanical solution of γ-FeMn and Ag) and finally increased the overall corrosion by inducing an enhanced micro-galvanic effect. XPS analyses after N-PIII proved the formation of mixed iron nitride and iron oxide on the plasma treated samples (PT). Electrochemical corrosion tests were performed in DPBSS and MHBSS. The corrosion rate of the MP sample (CR = 3.2 mm/year for MHBSS and 2.4 mm/year for DPBSS) was higher than the PT sample (2 for MHBSS and 1.9 mm/year for DPBSS). The lower corrosion rate of the PT sample referred to the desorption of Cl^−^ by N^δ−^ on the surface and the corrosion mechanism was discussed. A static degradation test was performed in MHBSS for 14 and 28 days. Then, after 14 days, there was a significant difference in the degradation rate of the MP and PT samples (0.24 and 0.18 mm/year, respectively), whereas after 28 days the degradation rates were close for both conditions (0.33 and 0.3 mm/year, respectively), which implied that the iron nitride layer was dissolved. The MTT assay confirmed a higher biocompatibility of the PT sample after 24, 72 and 120 h, according to the lower ion release after N-PIII. Hence, N-PIII could control the corrosion rate in the initial stages of degradation. So, an advanced absorbable Fe-based alloy with two stages of corrosion rate was effectively designed, the first stage, with a lower corrosion rate, and the second one with an accelerated rate, to reach a higher degradation. Further studies should be carried out to modify the processing parameters to prepare the desired degradation rate for biomedical applications. Furthermore, the hemocompatibility, carcinogenicity and in vivo tests should be carefully performed to study the complementary biological properties of the modified surfaces.

## Figures and Tables

**Table 1:** Ion concentration of human blood plasma, compared to the test solutions [33].

Ion or Compound (mg/L)	Blood Plasma	MHBSS	DPBSS
Na^+^	3000–3400	2795	3519
K^+^	130–210	172	162
Cl^−^	3400–3750	3542	4947
HCO_3_^−^	1100–2400	1654	-
H_2_PO_4_^−^/HPO_4_^2−^	270–450	48	920
Ca^2+^	84–110	35	-
Mg^2+^	15–30	14	-
SO_4_^2−^	5–15	78	-
D-Glucose	600–1100	720	-
Albumin	35,000–50,000	-	-

**Table 2:** Phase analysis and mechanical properties of the MP sample.

Composition (Wt.%)	Micro- Hardness (Hv), Phase	Hardness (HRB)	Yield Shear Strength (MPa)	Ultimate Shear Strength (MPa)	Ultimate Shear Strain (%)
Fe-30Mn-1Ag	103 ± 13, α-Fe	96.5 ± 3.1	361.5 ± 22.4	420.3 ± 35.1	74 ± 12
	556 ± 77, γ-FeMn				
	515 ± 91, α-Mn				

**Table 3:** MP and PT condition binding energy and FWHM values, percentage areas, Gaussian fraction and χ^2^ for C1s, O1s, N1s, Fe2p3 and Mn2p3.

Sample	Element	Peak1 (eV)	FWHM (eV)	Area (%)	Peak2 (eV)	FWHM (eV)	Area (%)	Peak3 (eV)	FWHM (eV)	Area (%)	Gauss (%)	χ^2^
MP	C	284.8	1.37	69.9	286.3	1.37	12.3	288.5	2.2	17.8	80	1.3
	O	529.8	1.4	37.1	531.7	1.8	54.7	533.3	2	8.2	80	1.3
	N	399.9	2.6	100	-	-	-	-	-	-	80	1.4
	Fe	708.5	2.7	20.1	710.2	2.5	42	712.2	3.2	37.9	90	1.2
	Mn	640.3	2.8	51.3	642.2	2.75	39.2	644.7	2.8	9.4	90	1.1
PT	C	284.8	1.5	85.7	286.5	1.1	4.8	288.4	1.86	9.5	70	1
	O	529.9	1.4	70.3	531.5	1.5	25.3	533.1	1.6	4.4	80	1.3
	N	396.2	1.8	100	403.6	2.9	100	-	-	-	80	0.9
	Fe	709.3	2.5	27.2	711	2.5	50.8	713.3	2.5	22.1	90	1.3
	Mn	640.4	2.8	44.3	642.1	2.8	39.5	644.7	2.8	16.2	90	1.3

**Table 4:** Electrochemical corrosion parameters derived from PDP (**A**) and EIS results (**B**). The meanings of the symbols are the following one: i_C_: corrosion current density; C_P_: corrosion potential; β_a_: anodic tafel constant; β_c_: cathodic tafel constant; CR: corrosion rate; R_1_: resistance of the porous outer oxide layer; Q_1_: capacitance of the porous outer oxide layer; n_1_: coefficient of Q_1_; R_2_: resistance value associated to the inner oxide/nitride layer; Q_2_: capacitance of the inner oxide/nitride layer; n_2_: coefficient of Q_2_; χ_ʋ_^2^: reduced χ^2^.

**(A) PDP Parameters**									
**Condition** **(Solution)**	**i_C_ (µA/cm^2^)**	**C_P_ (mV)**		**β_a_ (mV/decade)**		**β_c_ (mV/decade)**		**CR (mm/year)**	
MP + MHBSS	26.3 ± 4.1	−800 ± 77		116.7 ± 10		309.3 ± 27		3.2 ± 0.2	
MP + DPBSS	18 ± 16.5	−780 ± 49		132.4 ± 47.4		186.9 ± 60.9		2.4 ± 1.7	
PT + MHBSS	16 ± 11.3	−698 ± 96		141.5 ± 37		214.1 ± 40		2 ± 1.4	
PT + DPBSS	15.9 ± 2.2	−781 ± 81		123.2 ± 15		253.7 ± 32		1.9 ± 0.6	
**(B) EIS Parameters**									
**Condition** **(Solution)**	**R_1_ (ohm·cm^2^)**	**Q_1_** **(µF·cm^−2^·s^n^)**	**n_1_**	**τ_1_** **(s)**	**R_2_** **(ohm·cm^2^)**	**Q_2_** **(µF·cm^−2^·s^n^)**	**n_2_**	**τ_2_** **(s)**	**χ_ʋ_^2^**
MP + MHBSS	256 ± 9.9	115.7 ± 13	0.7 ± 0.1	0.03 ± 0.014	235.1 ± 11	220 ± 9.2	1 ± 0.1	0.027 ± 0.003	0.25
MP + DPBSS	754.7 ± 81.7	65.2 ± 7.6	0.7 ± 0.2	0.045 ± 0.006	311.1 ± 89.5	1454 ± 148	0.6 ± 0.1	0.02 ± 0.026	0.12
PT + MHBSS	673.4 ± 61	30.1 ± 5.9	0.9 ± 0.1	0.02 ± 0.005	1547 ± 578	946 ± 79	0.6 ± 0.3	1.46 ± 0.32	0.74
PT + DPBSS	349.3 ± 6.4	35.2 ± 2.4	0.7 ± 0.1	0.012 ± 0.002	921.6 ± 3.5	1637 ± 28.7	0.5 ± 0.1	1.51 ± 0.25	0.1

## Data Availability

All relevant data are contained in the present manuscript.

## References

[1] Hermawan H., Purnama A., Dube D., Couet J., Mantovani D. (2010). Fe-Mn alloys for metallic biodegradable stents: Degradation and cell viability studies. Acta Biomat..

[2] Hermawan H. (2012). Biodegradable Metals.

[3] Schinhammer M., Hanzi A.C., Loffler J.F., Uggowitzer P.J. (2010). Design strategy for biodegradable Fe-based alloys for medical applications. Acta Biomat..

[4] Witte F. (2010). The history of biodegradable magnesium implants: A review. Acta Biomat..

[5] Zheng Y.F., Gu X.N., Witte F. (2014). Biodegradable metals. Mat. Sci. Eng. R Rep..

[6] Li H., Zheng Y., Qin L. (2014). Progress of biodegradable metals. Progr. Nat. Sci. Mat. Intern..

[7] Vojtech D., Kubasek J., Serak J., Novak P. (2011). Mechanical and corrosion properties of newly developed biodegradable Zn-based alloys for bone fixation. Acta Biomat..

[8] Sotoudeh Bagha P., Khaleghpanah S., Sheibani S., Khakbiz M., Zakeri A. (2018). Characterization of nanostructured biodegradable Zn-Mn alloy synthesized by mechanical alloying. J. Alloys Compd..

[9] Sikora-Jasinska M., Paternoster C., Mostaed E., Tolouei R., Casati R., Vedani M., Mantovani D. (2017). Synthesis, mechanical properties and corrosion behavior of powder metallurgy processed Fe/Mg_2_Si composites for biodegradable implant applications. Mat. Sci. Eng. C.

[10] Li H., Lin G., Wang P., Huang J., Wen C. (2021). Nutrient alloying elements in biodegradable metals: A review. J. Mat. Chem. B.

[11] Otto M., Pilz S., Gebert A., Kühn U., Hufenbach J. (2021). Effect of Build Orientation on the Microstructure, Mechanical and Corrosion Properties of a Biodegradable High Manganese Steel Processed by Laser Powder Bed Fusion. Metals.

[12] Prokoshkin S., Pustov Y., Zhukova Y., Kadirov P., Karavaeva M., Prosviryakov A., Dubinskiy S. (2021). Effect of Thermomechanical Treatment on Structure and Functional Fatigue Characteristics of Biodegradable Fe-30Mn-5Si (wt %) Shape Memory Alloy. Materials.

[13] Wu W., Wang Z., Zang S., Yu X., Yang H., Chang S. (2020). Research Progress on Surface Treatments of Biodegradable Mg Alloys: A Review. ACS Omega.

[14] Zhu S., Huang N., Xu L., Zhang Y., Liu H., Sun H., Leng Y. (2009). Biocompatibility of pure iron: In vitro assessment of degradation kinetics and cytotoxicity on endothelial cells. Mat. Sci. Eng. C.

[15] Gasior G., Szczepanski J., Radtke A. (2021). Biodegradable Iron-Based Materials-What Was Done and What More Can Be Done?. Materials.

[16] Venezuela J., Dargusch M.S. (2020). Addressing the slow corrosion rate of biodegradable Fe-Mn: Current approaches and future trends. Curr. Op. Sol. State Mat. Sci..

[17] Hermawan H. (2018). Updates on the research and development of absorbable metals for biomedical applications. Prog. Biomater..

[18] Shuai C., Li S., Yang W., Yang Y., Deng Y., Gao C. (2020). MnO_2_ catalysis of oxygen reduction to accelerate the degradation of Fe-C composites for biomedical applications. Corr. Sci..

[19] Abdalla M., Joplin A., Elahinia M., Ibrahim H. (2020). Corrosion Modeling of Magnesium and Its Alloys for Biomedical Applications: Review. Corr. Mat. Degrad..

[20] Sotoudeh Bagha P., Khakbiz M., Sheibani S., Hermawan H. (2018). Design and characterization of nano and bimodal structured biodegradable Fe-Mn-Ag alloy with accelerated corrosion rate. J. Alloys Compd..

[21] Huang T., Cheng J., Zheng Y.F. (2014). In vitro degradation and biocompatibility of Fe-Pd and Fe-Pt composites fabricated by spark plasma sintering. Mat. Sci. Eng. C.

[22] Mandal S., Ummadi R., Bose M., Balla V.K., Roy M. (2019). Fe–Mn–Cu alloy as biodegradable material with enhanced antimicrobial properties. Mat. Lett..

[23] Thull R. (2010). Surface treatment. Metals for Biomedical Devices.

[24] Lu T., Qiao Y., Liu X. (2012). Surface modification of biomaterials using plasma immersion ion implantation and deposition. Interf. Focus.

[25] Cisternas M., Bhuyan H., Retamal M.J., Casanova-Morales N., Favre M., Volkmann U.G., Saikia P., Diaz-Droguett D.E., Mandl S., Manova D. (2020). Study of nitrogen implantation in Ti surface using plasma immersion ion implantation & deposition technique as biocompatible substrate for artificial membranes. Mater. Sci. Eng. C.

[26] Catanio Bortolan C., Paternoster C., Turgeon S., Paoletti C., Cabibbo M., Lecis N., Mantovani D. (2020). Plasma-immersion ion implantation surface oxidation on a cobalt-chromium alloy for biomedical applications. Biointerphases.

[27] Chen T., Castanon E., Gigax J.G., Kim H., Balerio R., Fan J., Garner F.A., Shao L. (2019). Nitrogen ion implantation into pure iron for formation of surface nitride layer. Nucl. Instr. Met. Phy. Res. B Beam Int. Mat. At..

[28] Bao J.J., Leng Y.X., Su Y.Y., Chen J.Y., Zhang P.C., Bai B., Huang N. (2011). High frequency and low voltage plasma immersion ion implantation of nitrogen on industrial pure iron at different Rf power. Surf. Coat. Technol..

[29] Bagha P.S., Khakbiz M., Sheibani S., Ebrahimi-Barough S., Hermawan H. (2020). In Vitro Degradation, Hemocompatibility, and Cytocompatibility of Nanostructured Absorbable Fe-Mn-Ag Alloys for Biomedical Application. ACS Biomat. Sci. Eng..

[30] Zhu S., Huang N., Xu L., Zhang Y., Liu H., Lei Y., Sun H., Yao Y. (2009). Biocompatibility of Fe–O films synthesized by plasma immersion ion implantation and deposition. Surf. Coat. Technol..

[31] Zhao Y., Wong S.M., Wong H.M., Wu S., Hu T., Yeung K.W., Chu P.K. (2013). Effects of carbon and nitrogen plasma immersion ion implantation on in vitro and in vivo biocompatibility of titanium alloy. ACS Appl. Mat. Interf..

[32] Nguyen H.G.T., Horn J.C., Bleakney M., Siderius D.W., Espinal L. (2019). Understanding material characteristics through signature traits from helium pycnometry. Langmuir.

[33] Tolouei R., Harrison J., Paternoster C., Turgeon S., Chevallier P., Mantovani D. (2016). The use of multiple pseudo-physiological solutions to simulate the degradation behavior of pure iron as a metallic resorbable implant: A surface-characterization study. Phys. Chem. Chem. Phys..

[34] (2014). Standard Test Method for Conducting Potentiodynamic Polarization Resistance Measurements.

[35] (2004). Standard Practice for Laboratory Immersion Corrosion Testing of Metals.

[36] (2012). Biological Evaluation of Medical Devices—Part 12: Sample Preparation and Reference Materials.

[37] Safaie N., Khakbiz M., Sheibani S., Bagha P.S. (2015). Synthesizing of Nanostructured Fe-Mn Alloys by Mechanical Alloying Process. Proc. Mat. Sci..

[38] (2013). Standard Hardness Conversion Tables for Metals Relationship among Brinell Hardness, Vickers Hardness, Rockwell Hardness, Superficial Hardness, Knoop Hardness, Scleroscope Hardness, and Leeb Hardness.

[39] Nieh T.G., Wadsworth J. (1991). Hall-petch relation in nanocrystalline solids. Scr. Metall. Et Mater..

[40] Wang Y.M., Ma E. (2004). Three strategies to achieve uniform tensile deformation in a nanostructured metal. Acta Mater..

[41] Alizadeh R., Mahmudi R., Langdon T.G. (2014). Superplasticity of a fine-grained Mg–9Gd–4Y–0.4Zr alloy evaluated using shear punch testing. J. Mat. Res. Technol..

[42] (2013). Standard Specification for Wrought 18Chromium-14Nickel-2.5Molybdenum Stainless Steel Bar and Wire for Surgical Implants (UNS S31673).

[43] Li Z., Tang G., Ma X., Sun M., Wang L. (2010). XPS Study on Chemical State and Phase Structure of PBII Nitriding M50 Steel. IEEE Trans. Plasma Sci..

[44] Graat P.C.J., Somers M.A.J., Mittemeijer E.J. (1998). The initial oxidation of ε-Fe_2_N_1−x_: An XPS investigation. Appl. Surf. Sci..

[45] Swift P. (1982). Adventitious carbon?the panacea for energy referencing?. Surf. Interface Anal..

[46] Miller D.J., Biesinger M.C., McIntyre N.S. (2002). Interactions of CO_2_ and CO at fractional atmosphere pressures with iron and iron oxide surfaces: One possible mechanism for surface contamination?. Surf. Interface Anal..

[47] Hueso J.L., Espinós J.P., Caballero A., Cotrino J., González-Elipe A.R. (2007). XPS investigation of the reaction of carbon with NO, O_2_, N_2_ and H_2_O plasmas. Carbon.

[48] Figueiredo J.L., Pereira M.F.R., Freitas M.M.A., Órfão J.J.M. (1999). Modification of the surface chemistry of activated carbons. Carbon.

[49] Liu Z., Duchon T., Wang H., Grinter D.C., Waluyo I., Zhou J., Liu Q., Jeong B., Crumlin E.J., Matolin V. (2016). Ambient pressure XPS and IRRAS investigation of ethanol steam reforming on Ni-CeO_2_(111) catalysts: An in situ study of C-C and O-H bond scission. Phys. Chem. Chem. Phys..

[50] Mullet M., Khare V., Ruby C. (2008). XPS study of Fe(II)–Fe(III) (oxy)hydroxycarbonate green rust compounds. Surf. Interface Anal..

[51] Biesinger M.C., Payne B.P., Grosvenor A.P., Lau L.W.M., Gerson A.R., Smart R.S.C. (2011). Resolving surface chemical states in XPS analysis of first row transition metals, oxides and hydroxides: Cr, Mn, Fe, Co and Ni. Appl. Surf. Sci..

[52] Yakupova I.V., Mamchenko A.V., Savchenko O.V., Chernova N.N., Kosygina I.M. (2016). Investigation of the structure of the surface of sorbents–catalysts modified with MnO2 by the method of X-ray photoelectronic spectroscopy. J. Water Chem. Technol..

[53] Heuer J.K., Stubbins J.F. (1999). An XPS characterization of FeCO_3_ films from CO_2_ corrosion. Corr. Sci..

[54] Diekmann W., Panzner G., Grabke H.J. (1989). The bonding state of nitrogen segregated on Fe(100) and on iron nitrides Fe_4_N and Fe_2_N. Surf. Sci..

[55] Liao H.M., Sodhi R.N.S., Coyle T.W. (1993). Surface composition of AlN powders studied by X-ray photoelectron spectroscopy and bremsstrahlung-excited Auger electron spectroscopy. J. Vac. Sci. Technol. A.

[56] Wu S.X., Xia Y.Q., Yu X.L., Liu Y.J., Li S.W. (2007). Magnetic properties of MnxTi_1−x_N thin films grown by plasma-assisted molecular beam epitaxy. J. Appl. Phys..

[57] Wang X., Zheng W.T., Tian H.W., Yu S.S., Xu W., Meng S.H., He X.D., Han J.C., Sun C.Q., Tay B.K. (2003). Growth, structural, and magnetic properties of iron nitride thin films deposited by dc magnetron sputtering. Appl. Surf. Sci..

[58] Rohith Vinod K., Saravanan P., Sakar M., Balakumar S. (2016). Insights into the nitridation of zero-valent iron nanoparticles for the facile synthesis of iron nitride nanoparticles. RSC Adv..

[59] Jie J., Shao T. (2017). Graded Microstructure and Mechanical Performance of Ti/N-Implanted M50 Steel with Polyenergy. Materials.

[60] Cui Q., Chao S., Wang P., Bai Z., Yan H., Wang K., Yang L. (2014). Fe–N/C catalysts synthesized by heat-treatment of iron triazine carboxylic acid derivative complex for oxygen reduction reaction. RSC Adv..

[61] Grosvenor A.P., Kobe B.A., Biesinger M.C., McIntyre N.S. (2004). Investigation of multiplet splitting of Fe 2p XPS spectra and bonding in iron compounds. Surf. Interface Anal..

[62] Wang X., Zheng W.T., Tian H.W., Yu S.S., Wang L.L. (2004). Effect of substrate temperature and bias voltage on DC magnetron sputtered Fe–N thin films. J. Magnet. Magn. Mat..

[63] Ram Mohan Rao K., Mukherjee S., Raole P.M., Manna I. (2002). Low energy isothermal plasma-immersion ion implantation of nitrogen for enhanced hardness of AISI 52100 ball bearing steel. Surf. Coat. Technol..

[64] Miola E.J., de Souza S.D., Nascente P.A.P., Olzon-Dionysio M., Olivieri C.A., Spinelli D. (1999). Surface characterisation of plasma-nitrided iron by X-ray photoelectron spectroscopy. Appl. Surf. Sci..

[65] Jutte R.H., Kooi B.J., Somers M.A.J., Mittemeijer E.J. (1997). On the oxidation ofα-Fe andε-Fe_2_N_1−z_: I. Oxidation kinetics and microstructural evolution of the oxide and nitride layers. Oxid. Met..

[66] Ilton E.S., Post J.E., Heaney P.J., Ling F.T., Kerisit S.N. (2016). XPS determination of Mn oxidation states in Mn (hydr)oxides. Appl. Surf. Sci..

[67] Liu Y., Xu L., Li X., Hu P., Li S. (2010). Growth and magnetic property of ζ-phase Mn2N_1±x_ thin films by plasma-assisted molecular beam epitaxy. J. Appl. Phy..

[68] Yang R., Yang L. (2018). Experimental Study of Static Contact-angle on Peak-like Microstructural Surfaces Produced by PIII Technology. J. Therm. Sci..

[69] Yao Z.Q., Yang P., Huang N., Sun H., Wang J. (2004). Structural, mechanical and hydrophobic properties of fluorine-doped diamond-like carbon films synthesized by plasma immersion ion implantation and deposition (PIII–D). Appl. Surf. Sci..

[70] Suegama P.H., de Melo H.G., Benedetti A.V., Aoki I.V. (2009). Influence of cerium (IV) ions on the mechanism of organosilane polymerization and on the improvement of its barrier properties. Electrochim. Acta.

[71] Hedberg Y., Karlsson M.E., Blomberg E., Odnevall Wallinder I., Hedberg J. (2014). Correlation between surface physicochemical properties and the release of iron from stainless steel AISI 304 in biological media. Coll. Surf. B Biointerf..

[72] Groth T., Altankov G. (1996). Studies on cell-biomaterial interaction: Role of tyrosine phosphorylation during fibroblast spreading on surfaces varying in wettability. Biomaterials.

[73] Mueller P.P., May T., Perz A., Hauser H., Peuster M. (2006). Control of smooth muscle cell proliferation by ferrous iron. Biomaterials.

[74] Nygren H. (1996). Initial reactions of whole blood with hydrophilic and hydrophobic titanium surfaces. Coll. Surf. B Biointerf..

[75] Hansen A.W., Führ L.T., Antonini L.M., Villarinho D.J., Marino C.E.B., Malfatti C.d.F. (2015). The Electrochemical Behavior of the NiTi Alloy in Different Simulated Body Fluids. Mat. Res..

[76] Jirásková Y., Brauer G., Schneeweiss O., Blawert C., Anwand W., Coleman P.G. (2002). The migration of defects and nitrogen atoms in nitrided surface layers of austenitic stainless steel followed by microscopic methods. Appl. Surf. Sci..

[77] Kim Y.-S., Kim D.-W., Lee I.-S., Yoon S., Kim D., Jun S., Cha B.-C. (2020). Effect of N+ Implantation on Surface Characteristics of 316L Stainless Steels for Bipolar Plate in PEMFC. Coatings.

[78] Ravi Kumar B., Singh R., Mahato B., De P.K., Bandyopadhyay N.R., Bhattacharya D.K. (2005). Effect of texture on corrosion behavior of AISI 304L stainless steel. Mat. Charact..

[79] Guleryuz L.F., Ipek R., Arıtman I., Karaoglu S. (2017). Microstructure and mechanical properties of Zn-Mg alloys as implant materials manufactured by powder metallurgy method. AIP Conf. Proc..

[80] Lee J.-S., Fushimi K., Nakanishi T., Hasegawa Y., Park Y.-S. (2014). Corrosion behaviour of ferrite and austenite phases on super duplex stainless steel in a modified green-death solution. Corr. Sci..

[81] Tao X., Liu X., Matthews A., Leyland A. (2019). The influence of stacking fault energy on plasticity mechanisms in triode-plasma nitrided austenitic stainless steels: Implications for the structure and stability of nitrogen-expanded austenite. Acta Mat..

[82] Ram Mohan Rao K., Mukherjee S., Roy S.K., Richter E., Möller W., Manna I. (2007). Plasma immersion ion implantation of nitrogen on austenitic stainless steel at variable energy for enhanced corrosion resistance. Surf. Coat. Technol..

[83] Fosbøl P.L., Thomsen K., Stenby E.H. (2013). Review and recommended thermodynamic properties of FeCO_3_. Corr. Eng. Sci. Technol..

[84] Mouzou E., Paternoster C., Tolouei R., Purnama A., Chevallier P., Dube D., Prima F., Mantovani D. (2016). In vitro degradation behavior of Fe-20 Mn-1.2C alloy in three different pseudo-physiological solutions. Mat. Sci. Eng. C.

[85] Martini E.M.A., Muller I.L. (2000). Characterization of the film formed on iron in borate solution by electrochemical impedance spectroscopy. Corr. Sci..

[86] Zhao C., Pan F., Zhao S., Pan H., Song K., Tang A. (2015). Preparation and characterization of as-extruded Mg–Sn alloys for orthopedic applications. Mat. Des..

[87] Naganuma H., Nakatani R., Endo Y., Kawamura Y., Yamamoto M. (2016). Magnetic and electrical properties of iron nitride films containing both amorphous matrices and nanocrystalline grains. Sci. Technol. Adv. Mat..

[88] Schibicheski Kurelo B.C.E., de Souza G.B., Serbena F.C., de Oliveira W.R., Marino C.E.B., Taminato L.A. (2018). Performance of nitrogen ion-implanted supermartensitic stainless steel in chlorine- and hydrogen-rich environments. Surf. Coat. Technol..

[89] Durai G., Kuppusami P., Maiyalagan T., Ahila M., Vinoth kumar P. (2019). Supercapacitive properties of manganese nitride thin film electrodes prepared by reactive magnetron sputtering: Effect of different electrolytes. Ceram. Intern..

[90] Zatkalikova V., Halanda J., Vana D., Uhricik M., Markovicova L., Strbak M., Kucharikova L. (2021). Corrosion Resistance of AISI 316L Stainless Steel Biomaterial after Plasma Immersion Ion Implantation of Nitrogen. Materials.

[91] Luiz L.A., Kurelo B.C.E.S., Souza G.B.d., Andrade J.d., Marino C.E.B. (2021). Effect of nitrogen plasma immersion ion implantation on the corrosion protection mechanisms of different stainless steels. Mater. Today Commun..

[92] Casero E., Parra-Alfambra A.M., Petit-Domínguez M.D., Pariente F., Lorenzo E., Alonso C. (2012). Differentiation between graphene oxide and reduced graphene by electrochemical impedance spectroscopy (EIS). Electrochem. Commun..

[93] Yohai L., Valcarce M.B., Vázquez M. (2016). Testing phosphate ions as corrosion inhibitors for construction steel in mortars. Electrochim. Acta.

[94] Grabke H.J. (1996). High Nitrogen Steels. The Role of Nitrogen in the Corrosion of Iron and Steels. ISIJ Intern..

[95] Tian X., Chu P.K. (2000). Electrochemical corrosion properties of AISI304 steel treated by low-temperature plasma immersion ion implantation. Scr. Mat..

[96] Baranwal P.K., Rajaraman P.V. (2019). Electrochemical investigation on effect of sodium thiosulfate (Na2S_2_O_3_) and ammonium chloride (NH_4_Cl) on carbon steel corrosion. J. Mat. Res. Technol..

[97] Gambaro S., Paternoster C., Occhionero B., Fiocchi J., Biffi C.A., Tuissi A., Mantovani D. (2021). Mechanical and degradation behavior of three Fe-Mn-C alloys for potential biomedical applications. Mat. Today Comm..

[98] Saravanan P., Raja V.S., Mukherjee S. (2007). Effect of plasma immersion ion implantation of nitrogen on the wear and corrosion behavior of 316LVM stainless steel. Surf. Coat. Technol..

[99] De Andrade L.M., Paternoster C., Montaño-Machado V., Barucca G., Sikora-Jasinska M., Tolouei R., Turgeon S., Mantovani D. (2018). Surface modification of L605 by oxygen plasma immersion ion implantation for biomedical applications. MRS Comm..

[100] Manova D., Höche T., Mändl S., Neumann H. (2009). Development of CrN precipitates during the initial stages of PIII nitriding of stainless steel thin films. Nucl. Instr. Meth. Phys. Res. Sec. B Beam Inter. Mat. At..

[101] Choe H.-C. (1999). Effects of nitrogen ion implantation on the corrosion characteristics of Cu-electroless plated and sintered stainless steel. Surf. Coat. Technol..

[102] Feng Q., Zhang D., Xin C., Liu X., Lin W., Zhang W., Chen S., Sun K. (2013). Characterization and in vivo evaluation of a bio-corrodible nitrided iron stent. J. Mat. Sci. Mat. Med..

[103] Zhao Y., Wu G., Jiang J., Wong H.M., Yeung K.W.K., Chu P.K. (2012). Improved corrosion resistance and cytocompatibility of magnesium alloy by two-stage cooling in thermal treatment. Corr. Sci..

[104] Yamamoto A., Kohyama Y., Kuroda D., Hanawa T. (2004). Cytocompatibility evaluation of Ni-free stainless steel manufactured by nitrogen adsorption treatment. Mat. Sci. Eng. C.

[105] Wan P., Ren Y., Zhang B., Yang K. (2010). Effect of nitrogen on blood compatibility of nickel-free high nitrogen stainless steel for biomaterial. Mat. Sci. Eng. C.

[106] Buhagiar J., Bell T., Sammons R., Dong H. (2011). Evaluation of the biocompatibility of S-phase layers on medical grade austenitic stainless steels. J. Mater. Sci. Mat. Med..

[107] Ali S., Abdul Rani A.M., Mufti R.A., Hastuty S., Hussain M., Shehzad N., Baig Z., Abdu Aliyu A.A. (2019). An Efficient Approach for Nitrogen Diffusion and Surface Nitriding of Boron-Titanium Modified Stainless Steel Alloy for Biomedical Applications. Metals.

[108] Braz J., Martins G.M., Sabino V., Vitoriano J.O., Barboza C.A.G., Soares A., Rocha H.A.O., Oliveira M.F., Alves Junior C., Moura C.E.B. (2019). Plasma nitriding under low temperature improves the endothelial cell biocompatibility of 316L stainless steel. Biotechn. Lett..

[109] Huang H.-H., Liu C.-F., Wang S., Chen C.-S., Chang J.-H. (2021). Nitrogen plasma immersion ion implantation treatment of Ti6Al7Nb alloy for bone-implant applications: Enhanced in vitro biological responses and in vivo initial bone-implant contact. Surf. Coat. Technol..

[110] Lau K., Heu C., Moore M.J., Zhang A., Akhavan B., Wise S.G., Bilek M.M.M., Lord M.S., Rnjak-Kovacina J. (2022). Effect of plasma ion immersion implantation on physiochemical and biological properties of silk towards creating a versatile biomaterial platform. Mater. Today Adv..

[111] Guo S., Liu N., Liu K., Li Y., Zhang W., Zhu B., Gu B., Wen N. (2020). Effects of carbon and nitrogen plasma immersion ion implantation on bioactivity of zirconia. RSC Adv..

[112] Huang T., Cheng Y., Zheng Y. (2016). In vitro studies on silver implanted pure iron by metal vapor vacuum arc technique. Coll. Surf. B Biointerf..

[113] Huang T., Cheng J., Bian D., Zheng Y. (2016). Fe-Au and Fe-Ag composites as candidates for biodegradable stent materials. J. Biomed. Mat. Res. B Appl. Biomat..

[114] Schinhammer M., Gerber I., Hanzi A.C., Uggowitzer P.J. (2013). On the cytocompatibility of biodegradable Fe-based alloys. Mater. Sci. Eng. C.

[115] Liu B., Zheng Y.F. (2011). Effects of alloying elements (Mn, Co, Al, W, Sn, B, C and S) on biodegradability and in vitro biocompatibility of pure iron. Acta Biomat..

